# Advancing diabetes prediction with a progressive self-transfer learning framework for discrete time series data

**DOI:** 10.1038/s41598-023-48463-0

**Published:** 2023-11-29

**Authors:** Heeryung Lim, Gihyeon Kim, Jang-Hwan Choi

**Affiliations:** 1 Division of Mechanical and Biomedical Engineering, Graduate Program in System Health Science and Engineering, Ewha Womans University, Seoul, 03760 Korea; 2 Department of Computational Medicine, Graduate Program in System Health Science and Engineering, Ewha Womans University, Seoul, 03760 Korea

**Keywords:** Computational biology and bioinformatics, Health care

## Abstract

Although diabetes mellitus is a complex and pervasive disease, most studies to date have focused on individual features, rather than considering the complexities of multivariate, multi-instance, and time-series data. In this study, we developed a novel diabetes prediction model that incorporates these complex data types. We applied advanced techniques of data imputation (bidirectional recurrent imputation for time series; BRITS) and feature selection (the least absolute shrinkage and selection operator; LASSO). Additionally, we utilized self-supervised algorithms and transfer learning to address the common issues with medical datasets, such as irregular data collection and sparsity. We also proposed a novel approach for discrete time-series data preprocessing, utilizing both shifting and rolling time windows and modifying time resolution. Our study evaluated the performance of a progressive self-transfer network for predicting diabetes, which demonstrated a significant improvement in metrics compared to non-progressive and single self-transfer prediction tasks, particularly in AUC, recall, and F1 score. These findings suggest that the proposed approach can mitigate accumulated errors and reflect temporal information, making it an effective tool for accurate diagnosis and disease management. In summary, our study highlights the importance of considering the complexities of multivariate, multi-instance, and time-series data in diabetes prediction.

## Introduction

Diabetes mellitus is a significant global health issue. Approximately one in 11 adults are affected worldwide, and 90% of the cases are type 2 diabetes mellitus (T2DM)^1^. Diabetes-associated mortality rates have increased by 3% across all age groups from 2000 to 2019, as reported by the World Health Organization (WHO)^2^. Diabetes and its complications are leading causes of disability and mortality. Early diagnosis is crucial for effective disease management and improving the life quality of the patients^3^. Accordingly, several trials have been conducted to predict the development of diabetes accurately^4–6^.

Despite the multifactorial nature of diabetes development, few studies have focused on predicting diabetes using multivariate, multi-instance, and time series data. The irregular visit patterns of patients (varying frequency and stay length) and the diversity of individual pathologies make collecting and organizing time series data challenging^7^. Statistical methods such as time series regression and dimension reduction have traditionally been used for disease prediction^8^. However, the recent development of deep learning algorithms has enabled the application of deep neural architectures in diverse research tasks, including medical data with high correlations and dimensions^9^. For instance, the first investigation of diabetes multivariate time series prediction using deep learning models^7^ introduced the possibility of applying long short-term memory (LSTM) and gated-recurrent unit (GRU) on clinical data. The PIMA Indian dataset (PID), provided by the National Institute of Diabetes and Digestive and Kidney Diseases (NIDDK), is one of the most widely used datasets for diabetes prediction. This is because the dataset has a high prevalence of diabetic outbreaks and includes several important features. Various approaches, including artificial neural networks (ANN), naive bayes (NB), decision trees (DT), and deep learning (DL), have been explored to provide an effective prognostic tool for healthcare professionals^5^. Recent approaches have successfully incorporated CNN, CNN-LSTM, and CNN-BiLSTM, significantly enhancing the metrics on a large scale^10,11^. Most studies on the Korean Genome and Epidemiology Study (KoGES) dataset have focused on identifying correlations between certain factors and diabetes development using statistical methods^12–15^. These studies have demonstrated correlations between diabetes development and factors such as waist circumference^12^, prehypertension, hypertension, and glycated hemoglobin levels^13^. However, the only application of time series prediction of diabetes using deep learning models based on the KoGES dataset thus far has been limited to a vanilla LSTM model that does not reflect the characteristics of this data in its structure^16^. Additionally, there is a need for a novel method that can enhance disease prediction in more imbalanced datasets with a significantly larger number of features and instances. Therefore, we have developed a sophisticated deep learning framework that can detect dynamic temporal patterns in feature combinations and label properties, enhancing diabetes development prediction performance. This framework includes adequate data preprocessing methods, such as feature selection and data imputation. Using the KoGES Ansan and Ansung dataset, we aimed to improve diabetes prediction using multivariate, multi-instance, and time series data.

Time series analysis is a commonly used technique in various fields where the collected data has temporal dimensions. In such cases, time series classification frequently benefits from the enhancement of convolutional neural networks (CNNs)^17,18^. Unlike conventional statistical methods relying on variance distribution, such as correlation analysis^8^, deep learning algorithms have been developed and applied successfully to time series analysis after the recent advances in artificial intelligence (AI)^9^. Disease prediction is a domain where time series analysis has been applied, targeting specific features or the overall condition of the patient. Active research related to chronic diseases including diabetes and hypertension has been conducted due to the growing size of the affected populations and the social interest in these conditions. For example, recent studies have addressed the application of deep neural networks (DNNs) in hypertension^19^, as well as the use of LSTM and multi-layer perceptrons (MLPs) in heart disease^20^. Furthermore, the recent Corona virus 2019 (COVID-19) epidemic has led to various time series prediction tasks, including the use of deep learning algorithms such as LSTM, GRU and bi-LSTM^21,22^. Since many researchers continue to focus on data engineering and the optimization of existing models, there should be attempts to develop more sophisticated deep learning frameworks for novel insights. Meanwhile, recent research in time series prediction in the medical domain has focused on self-supervised algorithms to overcome the problems associated with inadequately labeled and incompletely collected data. These algorithms aim to capture temporal dynamics and enable early intervention for patients^23^. However, inherent difficulties in detecting the progression of associated features still pose problems, including multiple covariates, progression heterogeneity, and data storage issues^24^. Despite these challenges, time series prediction research continues to advance in the medical field, with potential for significant improvements in disease diagnosis and management.

Transfer learning is a methodology used to convey information across data in neural networks. The three major approaches of developing the model algorithm scheme are what, how, and when to transfer, with the selection of information boundaries for the target task, knowledge transfer, and tuning methods, such as pruning and layer freezing, determining transfer learning performance^25^. Therefore, careful consideration of the details of transfer learning implementation is necessary. One of the methods of transfer learning involves model weight initialization, where the knowledge acquired from the source domain is transferred to the target domain by initializing model weights, alleviating the performance of the target task. Recent research in transfer learning investigates diverse forms of datasets, including time series data, image data, and text data^26–28^. In time series prediction, studies have focused on multimodal data, multitask learning, and self-supervised approaches for the informative fusion of available datasets, providing appropriate task results^28,29^. Additionally, researchers have explored the selection of appropriate source domains among diverse datasets to overcome problems frequently encountered in time series datasets, such as missing labels^30^. However, shortages of large general datasets remain a challenge for future studies. Humans perceive most time series data not only sequentially but also as a whole. Motivated by this idea, the application of transfer learning and self-supervised learning in this study focuses on ongoing temporal self-data addition and the implementation of the idea into the model structure, where the data is in the same feature space. This approach is expected to improve the performance of time series prediction models in various applications.

Here, we present a novel approach to time series disease prediction by adjusting time series data through the modification of time windows and time resolutions in a manner similar to data masking in self-supervised learning. Our proposed model framework transfers information, including the unseen patterns of variables and the temporal properties of labels, to predict diabetes development in each individual. We also apply ensemble techniques to calibrate multiple learners, demonstrating the potential applications of AI tools in the early prediction of diabetes. Our contributions include:

- The introduction of a novel progressive self-transfer framework for time series disease prediction, which effectively teaches dynamic temporal patterns via downstream classifiers.
- The introduction of efficacious methods to process discrete time series data, such as shifting and rolling window and modifying time resolution. By doing so, the total number of model training to learn important representative features was increased.
- Extensive training and evaluation of our method using a large dataset that is multivariate, multi-instance, and in time series. Given its ability to handle diverse datasets beyond our current study, our approach has potential extensibility.

Overall, our proposed approach has significant potential to improve the accuracy of diabetes prediction and may have broader implications for other time series prediction tasks in the medical domain.

## Results

Results are presented in the order of experimental complexity and in the order in which they were conducted. We begin by presenting the results of the baseline experiments, which serve as a reference for comparison with the proposed downstream classifiers. Following this, we present the results of the single progressive self-transfer networks, which function as downstream classifiers. Finally, we introduce the ensemble results, which combine multiple classifiers.

### Non-progressive self-transfer network

Non-progressive self-transfer networks are used as a baseline model for comparison before estimating the performance of each progressive self-transfer network. In this study, we introduce four downstream classifiers, called submodels, and the four non-progressive self-transfer networks are used to predict whether each patient, distinguished by their own code, experiences diabetes at the last time-step of the dataset. Despite having data for every time-step, we preprocessed the data dimensions to match the final prediction task of the four single progressive self-transfer networks. The descriptions of the four non-progressive baseline predictions, which are the output of the last time-step of the dataset and serve as the baseline output of the four progressive self-transfer networks, are shown in Table 1. Our evaluation of the baseline models revealed that, in four of the five evaluation categories (accuracy, AUC, precision, and F1 score), the best performance was observed when most of the data was used for training. However, we also observed that the best recall was obtained when only two time steps were used, indicating that the rest of the baseline model did not show a significant performance loss.

**Table 1:** Ten iterations of the four non-progressive self-transfer models results. *t*_*n*_, the name of the time-step in every two years; *t*_*7*_, the data collected from 2017 to 2018, the label of which is to be predicted. AUC, area under the curve. Data are reported as the mean** ± **SD.

Data	Prediction	Accuracy	AUC	Recall	Precision	F1 score
*t*_*1*_*–t*_*6*_	*t*_*7*_	**83.85 ± 1.35**	**92.07 ± 0.08**	84.49 ± 2.59	**51.17 ± 2.39**	**63.37 ± 1.34**
*t*_*4*_*–t*_*6*_	*t*_*7*_	82.76 ± 1.38	91.65 ± 0.07	84.45 ± 2.58	49.48 ± 2.20	61.88 ± 1.11
*t*_*2*_*, t*_*4*_*, t*_*6*_	*t*_*7*_	82.35 ± 0.84	91.16 ± 0.13	84.47 ± 2.14	48.36 ± 1.48	61.17 ± 0.80
*t*_*3*_*, t*_*6*_	*t*_*7*_	81.30 ± 1.22	90.76 ± 0.12	**85.34 ± 1.67**	46.78 ± 1.75	60.10 ± 1.17

The highest performance values for each evaluation metric are indicated in bold.

### Single progressive self-transfer network

In this stage of our study, we applied progressive self-transfer learning to our model for each task in sequence. Each network had its own first task, which was designed based on the time series data preprocessing methods. These first tasks served as a foundation for the sequential learning tasks that followed. In this sequential stage, the knowledge from the previous learning step was transferred to the next learning step through weight initialization. This approach allowed the transfer of information in a progressive manner over time, resulting in what we call progressive self-transfer networks. Table 2 displays the results of the single progressive self-transfer networks. Our progression approach was implemented using two methods: (1) shifting and rolling window and (2) modifying time resolution. The first two models were designed using the shifting and rolling window method, while the next two models were preprocessed using the method of modifying time resolution. We found that the results of submodel 1 were significantly better than those of the other models in four of the five evaluation metrics. However, the evaluation performance of the other models also showed similar superiority. Additionally, comparing these results to those in Table 1, we observed a progression in performance in most of the evaluation metrics, especially in accuracy, area under the curve (AUC), and recall.

**Table 2:** Ten iterations of the four single progressive self-transfer models results. Submodel 1, submodel 2, submodel 3, and submodel 4 are the downstream classifiers. The best performance in each evaluation metric among single progressive self-transfer networks is indicated in bold. Data are reported as the mean** ± **SD.

	Accuracy	AUC	Recall	Precision	F1 score
Submodel 1	**83.66 ± 0.83**	**92.07 ± 0.08**	84.56 ± 1.16	**50.68 ± 1.33**	**63.07 ± 0.97**
Submodel 2	83.32 ± 0.84	91.75 ± 0.13	85.49 ± 1.99	49.98 ± 1.55	62.81 ± 0.77
Submodel 3	81.50 ± 1.08	91.18 ± 0.16	**85.58 ± 1.36**	46.97 ± 1.44	60.36 ± 1.03
Submodel 4	81.38 ± 0.66	90.94 ± 0.13	85.51 ± 1.63	46.65 ± 0.83	60.14 ± 0.51

### Multiple progressive self-transfer ensemble network

The final stage of our study involved the application of ensemble techniques to integrate the results of the single progressive self-transfer networks. As we had four different networks (submodel 1–4), we experimented with every possible combination of ensemble networks to identify the trends in the results. Submodel 1 and submodel 2 were downstream classifiers that processed time series data using the shifting and rolling window method, while submodel 3 and submodel 4 were downstream classifiers that processed time series data by doubling and tripling the time resolution.

**Figure 1:**
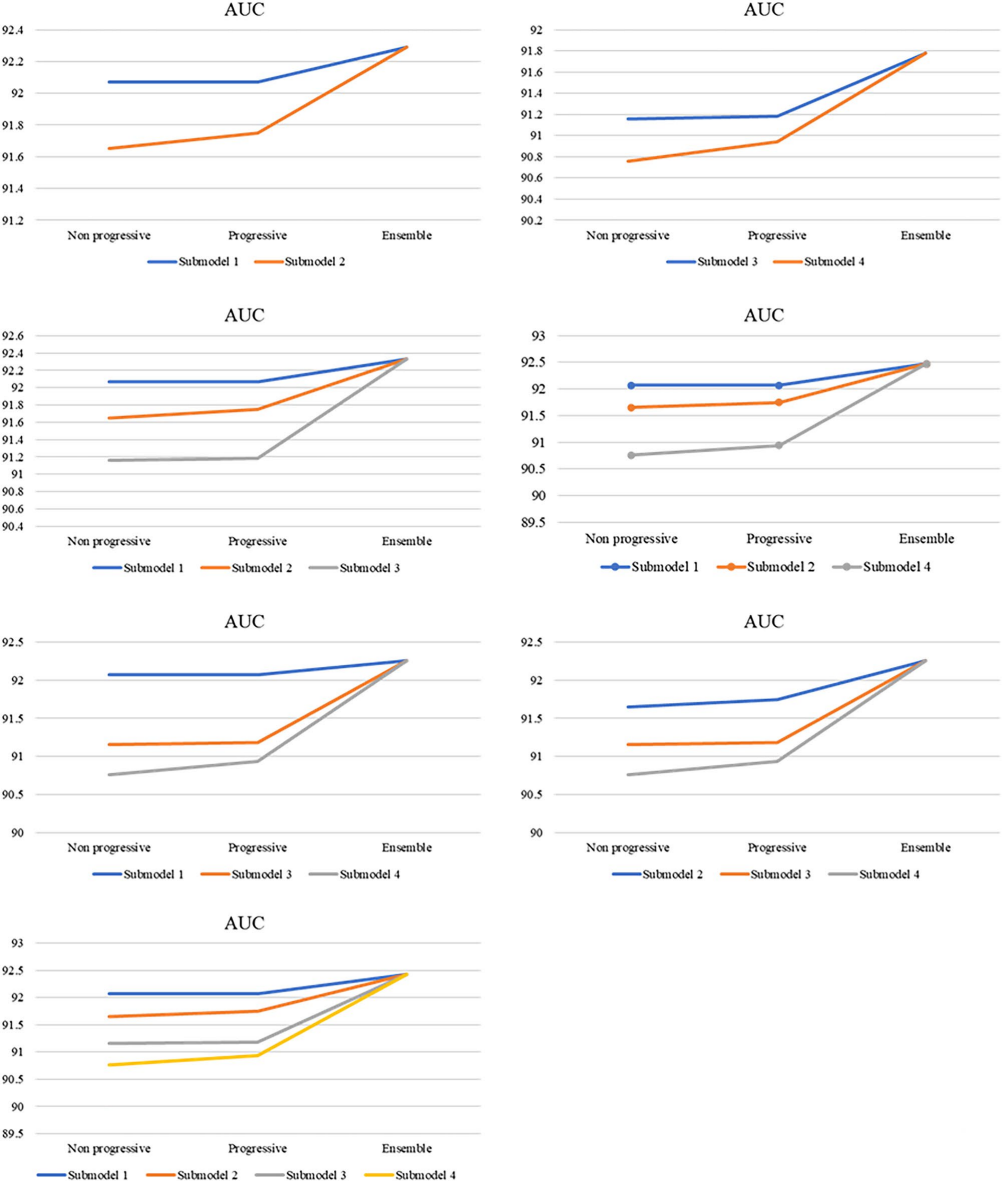
Trends in the evaluation metrics, particularly the AUC, are depicted for all experiments described.

Table 3 shows the ensemble results of every combination of the four models among single progressive self-transfer networks. The first two rows display the results of the ensemble for only two downstream classifiers. The experiments were conducted for only submodel 1 with submodel 2 and submodel 3 with submodel 4, based on the time series data processing method used. The next four rows show the ensemble results of the three downstream classifiers. AUC, recall, and F1 score indicated improved performance in all ensemble results compared to the single downstream classifier results described in Table 2. The last row displays the ensemble results of all four downstream classifiers. Four of the five evaluation metrics outperformed the results of all four downstream classifiers, except for precision. Furthermore, Fig. 1 illustrates the schema of all experimental cases described in Table 3. This figure focuses on AUC, which served as our criterion for determining the best epoch of each stage.

**Table 3:** Ten iterations of the ensemble results. Each result is from the multiple progressive self-transfer network, which consists of downstream classifiers described. The best AUC performance among ensemble results is indicated in bold. Data are reported as the mean** ± **SD.

	Downstream classifiers	Accuracy	AUC	Recall	Precision	F1 score
2	Submodel 1, Submodel 2	84.15 ± 0.35	92.29 ± 0.10	85.30 ± 1.16	51.29 ± 0.61	63.90 ± 0.38
	Submodel 3, Submodel 4	82.28 ± 0.73	91.78 ± 0.09	86.30 ± 1.97	48.02 ± 1.09	61.52 ± 0.79
3	Submodel 1, Submodel 2, Submodel 3	83.68 ± 0.28	92.33 ± 0.08	85.64 ± 0.86	50.32 ± 0.51	63.27 ± 0.32
	Submodel 1, Submodel 2, Submodel 4	83.99 ± 0.40	**92.47 ± 0.07**	86.07 ± 0.60	50.93 ± 0.71	63.86 ± 0.52
	Submodel 1, Submodel 3, Submodel 4	83.46 ± 0.65	92.26 ± 0.06	86.48 ± 0.85	49.95 ± 1.08	63.18 ± 0.75
	Submodel 2, Submodel 3, Submodel 4	83.21 ± 0.36	92.26 ± 0.07	86.53 ± 1.13	49.46 ± 0.54	62.82 ± 0.33
4	Submodel 1, Submodel 2, Submodel 3, Submodel 4	83.71 ± 0.35	92.43 ± 0.04	86.30 ± 0.64	50.35 ± 0.57	63.48 ± 0.42

We found that the combination of submodel 1, submodel 2, and submodel 4 produced the best performance in terms of AUC and other metrics overall. Thus, we conducted additional experiments to thoroughly inspect the validity of the combinations. Table 4 shows the ensemble results of every possible combination among submodel 1, submodel 2, and submodel 4. The best performance in AUC was obtained with the combination of submodel 1, submodel 2, and submodel 4, which outperformed the ensemble results of all four downstream classifiers in Table 3.

**Table 4:** Ten iterations of the ensemble results of the combinations of submodels 1, 2, and 4. The best AUC performance among ensemble results is indicated in bold. Data are reported as the mean** ± **SD.

	Downstream classifiers	Accuracy	AUC	Recall	Precision	F1 score
2	Submodel 1, Submodel 2	84.15 ± 0.35	92.29 ± 0.10	85.30 ± 1.16	51.29 ± 0.61	63.90 ± 0.38
	Submodel 1, Submodel 4	83.70 ± 0.76	92.21 ± 0.11	86.08 ± 0.72	50.51 ± 1.21	63.49 ± 0.88
	Submodel 2, Submodel 4	83.39 ± 0.48	92.22 ± 0.10	86.54 ± 1.09	49.82 ± 0.80	63.10 ± 0.50
3	Submodel 1, Submodel 2, Submodel 4	83.99 ± 0.40	**92.47 ± 0.07**	86.07 ± 0.60	50.93 ± 0.71	63.86 ± 0.52

Furthermore, the experimental results were compared to the baseline models: LSTM, GRU, and RNN. Table 5 displays the performance metrics obtained from each case. The first three rows present the metrics of the baseline models, which utilized the same dataset and hyperparameter settings as the proposed model for comparison. The last row showcases the metrics of the proposed model, referring to the combination of submodel 1, submodel 2, and submodel 4, which exhibited the best AUC performance as shown in Table 3. Overall, the proposed model outperformed all the baseline models in terms of AUC and recall. Considering the highly imbalanced label ratio, we can observe an improvement in the ability to detect diabetes patients on a larger scale. Additionally, given that our proposed model is based on a multilayered LSTM, which demonstrates the best AUC among the baseline models, we can also see that the model exhibits similar patterns in accuracy, precision, and F1 score when compared to the baseline LSTM model.

**Table 5:** Ten iterations of the baseline models and comparison with the proposed model. The best performance in each evaluation metric among single progressive self-transfer networks is indicated in bold. Data are reported as the mean** ± **SD.

	Accuracy	AUC	Recall	Precision	F1 score
LSTM	83.85 ± 1.35	92.07 ± 0.08	84.49 ± 2.59	51.17 ± 2.39	63.37 ± 1.34
GRU	**87.72 ± 0.51**	91.63 ± 0.10	73.19 ± 2.64	**61.00 ± 1.72**	**66.00 ± 0.84**
RNN	87.49 ± 0.41	91.62 ± 0.08	73.35 ± 1.81	60.21 ± 1.47	65.63 ± 0.80
Proposed model	83.99 ± 0.40	**92.47 ± 0.07**	**86.07 ± 0.60**	50.93 ± 0.71	63.86 ± 0.52

**Figure 2:**
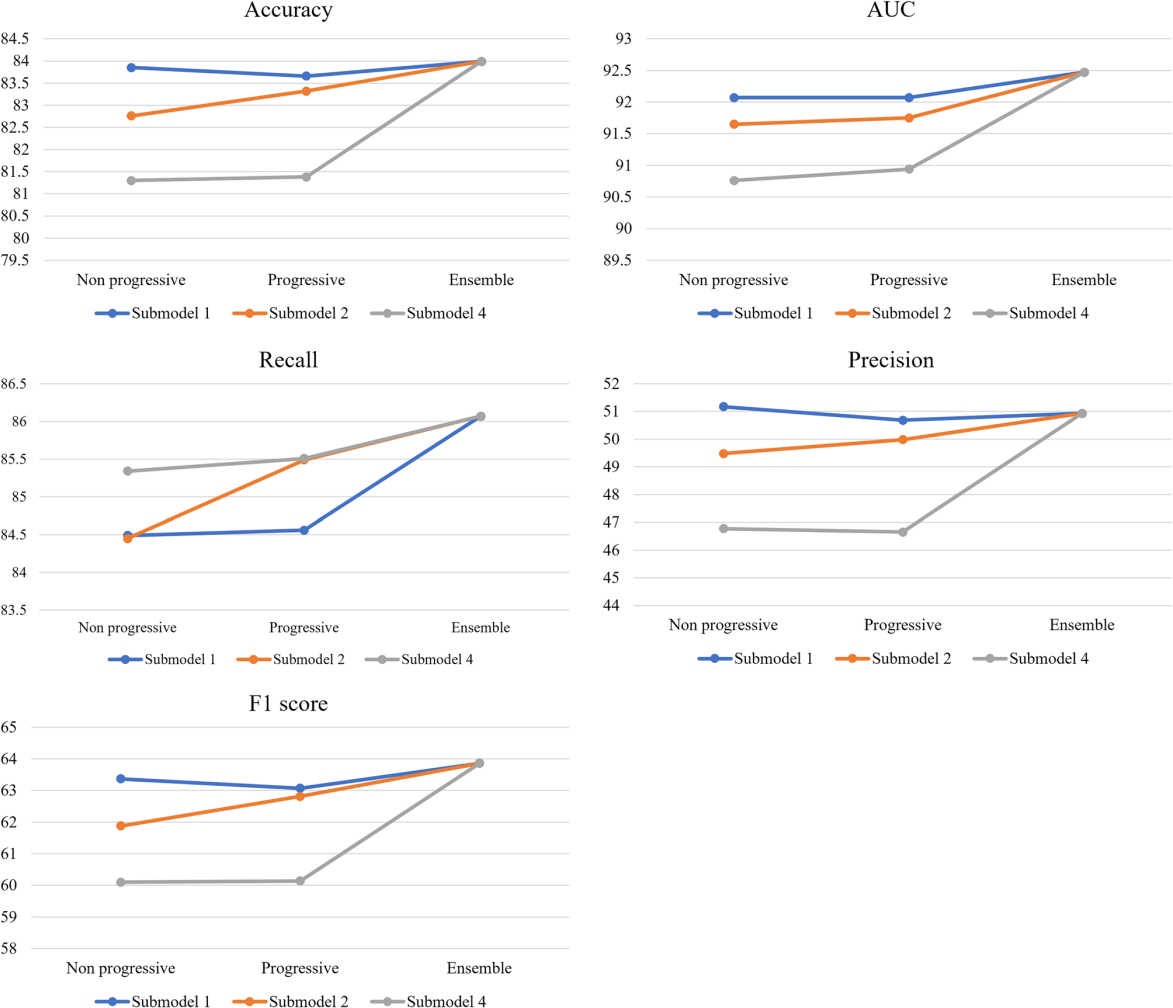
Trends in the evaluation metrics. Accuracy, AUC, precision, recall, and F1 score for non-progressive self-transfer models, single progressive self-transfer networks, and ensemble results of submodel 1, submodel 2, and submodel 4 are descripted.

The performance of the proposed model is presented in Fig. 2. A gradual improvement in model performance was noted across the evaluation metrics, highlighting the effectiveness of the proposed approach. Furthermore, the interactions between the single progressive self-transfer networks were also observed. For instance, the accuracy of submodel 4 improved significantly, surpassing submodels 1 and 2. The ensemble approach enhanced the accuracy of all four submodels, particularly that of submodel 4. Additionally, an increase in recall is crucial in medical domains as it prevents missing potential patients, and the proposed model showed promising results in improving recall.

## Discussion

Given the complexity of the disease and unknown interactions between related factors, the accurate prediction of diabetes development is crucial. In this study, we proposed a progressive self-transfer network that incorporates time series data preprocessing methods, shifting and rolling window and modifying time resolution, to reflect feature representations in multivariate and multi-instance time series analysis. The proposed method also accounts for dynamic temporal patterns, including temporal imbalance of the label, which is common in medical data. The gradual improvement of the metrics performances shown in Fig. 2 indicates that the progressive self-transfer network followed by ensemble method efficiently integrates and employs information added over time, enabling each downstream classifier to interpret the dataset from their own perspective. The results demonstrate that the proposed method can effectively recognize previously unseen data patterns and transfer the acquired knowledge as background information to the sequential tasks. Therefore, the model can now detect more patients earlier than before, enabling early diagnosis and intervention.

Furthermore, our study contributes to the field by utilizing deep learning methods for time series prediction tasks on the KoGES dataset. To the best of our knowledge, there have been limited studies on this dataset using deep learning techniques. Previous studies have used conventional algorithms and primarily focused on identifying the association of a single or a few factors with the development of diabetes. In contrast, our approach considers multiple relevant features to predict diabetes development, providing a more comprehensive understanding of the disease. As such, our study offers a significant contribution to the field of diabetes prediction using time series analysis and deep learning methods.

In order to optimize the LSTM model used in this research, we conducted a series of experiments adjusting various parameters. These experiments were performed across all stages, including the non-progressive self-transfer networks, single progressive self-transfer networks, and their ensemble applications. We tested LSTM models with different numbers of layers, and found that the model with five layers performed slightly better than the four-layer model in some metrics, but with a significant increase in standard deviations. We also manually adjusted the dropout rate and input unit size. Additionally, we optimized the methods used to modify the time series data, including the initial window size and the expansion of time intervals. Since we had a limited number of discrete time steps, we chose to double or triple the time resolution, which allowed us to finalize the structure of the input and output data.

It is important to note that our study may have potential bias or human error issues, which can arise in discriminative supervised models. One potential source of human error is in the labeling process, which can be influenced by misreported survey responses or biases introduced during clinical measurements. Moreover, our study did not discriminate between type 1 diabetes mellitus (T1DM) and T2DM, which limits our understanding of the participants’ diabetes mellitus development. To address these limitations, future studies could focus on disease-specific data collection to improve the reliability of the labels and allow the discrimination between T1DM and T2DM. Additionally, incorporating genetic information such as single nucleotide polymorphisms (SNPs) could enhance the model’s background knowledge and enable personalized patient care. SNPs, in combination with lifestyle habits, could serve as key factors in diabetes development and support more effective patient interventions.

## Conclusions

In conclusion, our study presents a novel progressive self-transfer learning network that integrates information over time to predict diabetes development at a target time-step with remarkable performance. Our method has several advantages, including mitigating the problem of accumulated error in recursive neural networks, predicting multiple timepoints and utilizing each result in sequence, and improving the learning of important representative features through the modification of time series data. Our findings have implications for the field of digital healthcare for chronic diseases, particularly in the potential of AI to improve clinical efficiency and aid in early diagnosis and intervention for diseases like diabetes mellitus. Indeed, as the proposed model helps to detect more patients with improved metrics, our findings can significantly enhance the opportunity for early detection. With these promising results, our method can contribute to the development of digital healthcare and preventative medicine, enhancing the expertise of healthcare providers and improving the health outcomes of patients.

## Methods

### The Korean Genome and Epidemiology Study

The Korean Genome and Epidemiology Study (KoGES) Ansan and Ansung dataset was used in this research. The KoGES consortium aims to investigate the genetic–environmental factors and interactions in common and complex diseases in Koreans. The study is an ongoing community-based cohort study conducted by the Korea Disease Control and Prevention Agency (KDCA) and the Ministry of Health and Welfare. The dataset contains biannual medical checkup and survey data of participants (40–69 years old) from 2001 to 2018, residing in either urban (Ansan) or rural (Ansung) areas. The cohort baseline with 10,030 participants was established in 2001–2002, and 6157 participants attended the last time-step in 2017–2018. The objective of the study was to predict whether each participant would develop diabetes at the last time-step. The American Diabetes Association (ADA) guidelines were followed, and a participant was considered to have diabetes if they fulfilled at least one of the criteria listed in Table 6. In other words, labels for each time-step were generated by considering both inspection features, which are based on ADA criteria, and survey features, which strongly indicate whether the participant was diagnosed with diabetes in the preceding 2 years. The label creation is based on the assumption that data at a specific time step influences subsequent diabetes cases after two years, aligning with the interval of the KoGES dataset.

**Table 6:** Criteria for diabetes development. OGTT, oral glucose tolerance test.

Selected Attribute		Criterion	Remark
Inspection	HbA1C	> = 6.5%	Whole blood
	Fasting Glucose	> = 126 mg/dL	Plasma
	Glucose OGTT (120 min)	> = 200 mg/dL	Plasma
Survey	Experience of Diabetes diagnosis	Yes	Last 2 years
	Ongoing treatment of Diabetes	Yes	Last 2 years
	Ongoing insulin therapy	Yes	Last 2 years

Table 7 presents the labels used in the study for each time-step, where “label 1” indicates diabetic participants and “label 0” indicates non-diabetic participants. The labels were generated based on specific criteria as described in Table 6 and were inferred from the data collected at each time-step *t*. Out of the 3995 participants who participated in every time-step, 3379 were included in the proposed network analysis after excluding those who already had diabetes at the first time-step. Table S1 provides a detailed description of the data used in the study. Notably, the ratio of diabetic (label 1) to non-diabetic (label 0) participants shows a gradual increase (Table 7), more than quadrupling from the first to the last time-step. Furthermore, the proposed framework utilized *t*_*n-1*_ data to generate a label for *t*_*n*_ data for early prediction of the next time-step and used the latest six time-step sets of the data as an input for the proposed framework.

**Table 7:** Label information of every time-step. Label at *t*_*n*_ refers to the diabetes development information at the time-step *t*_*n*_. For instance, the column *t*_*1*_ lists the label information inferred by the data of 2005–2006 and the column *t*_*2*_ by that of 2007–2008. The numbers, except those in the last row, indicate number of people.

	*t*_*1*_	*t*_*2*_	*t*_*3*_	*t*_*4*_	*t*_*5*_	*t*_*6*_	*t*_*7*_
Label 0 (non-diabetic)	3249	3180	3111	3076	3026	2939	2824
Label 1 (diabetic)	130	199	268	303	353	440	555
Label 1 (%)	3.85	5.89	7.93	8.97	10.45	13.02	16.42

### LASSO feature selection

Feature selection is a crucial step in network training to ensure optimal model performance and efficiency^31^. In this study, we employed the least absolute shrinkage and selection operator (LASSO) feature selection to identify the most relevant features for the proposed classification task. First, we sorted all variables and participants in every time-step, resulting in a dataset of 3379 participants and 850 features. We then selected only continuous features with less than 80% missing values in each time-step, resulting in a final dataset of 3379 participants and 56 features. The missing values in the dataset were imputed using bidirectional recurrent imputation for time series (BRITS). LASSO feature selection was then applied to the final dataset to select the most relevant features for the network training process.

**Figure 3:**
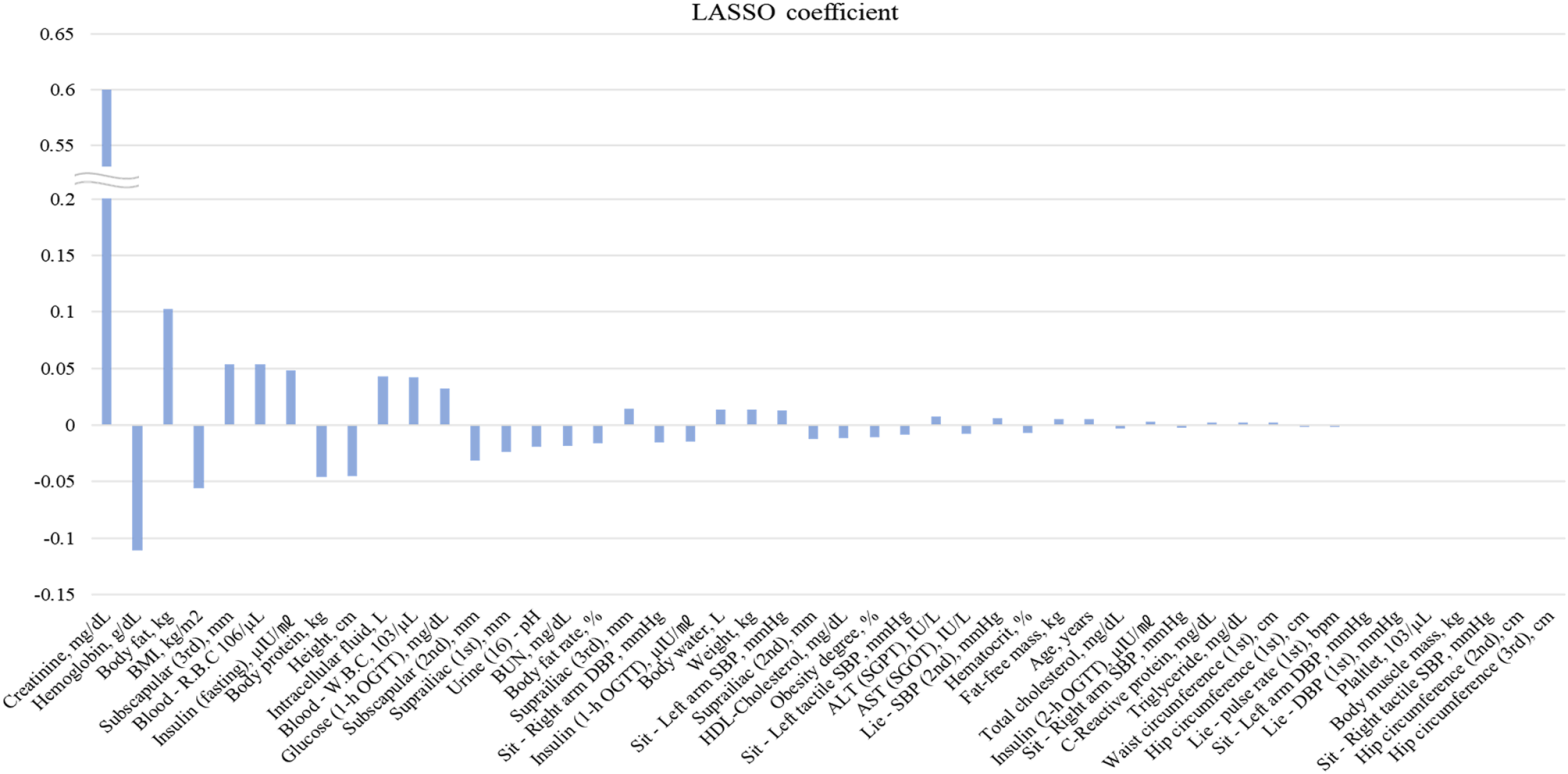
Diagram of the LASSO coefficients of the selected 48 features. The coefficients are sorted in descending order based on the absolute value.

After performing basic preprocessing steps, LASSO was adapted for feature selection. LASSO is a type of regularized linear regression that controls the penalty strength to shrink insignificant coefficients to zero, reducing dimensionality and minimizing the number of features relevant to the labels simultaneously^32^. Grid search was applied to find the most suitable penalty coefficient, resulting in the selection of 48 features with positive or negative correlation to each label data. The coefficients of the 48 selected features are displayed in Fig. 3**.** Creatinine in blood serum, hemoglobin in whole blood, body fat, body mass index (BMI), and subscapular measurement (3^rd^) were identified as the top five features with the largest coefficients in magnitude. The larger the coefficient magnitude, the more effective the explanatory variables^33^. Additionally, the sign of the coefficient indicates a positive or a negative correlation with the label data^33^. The total demographic information of the selected 48 features is described as descriptive statistics in Table S1, and the detailed information of the features with LASSO coefficients is shown in Table S2.

### BRITS imputation

**Figure 4:**
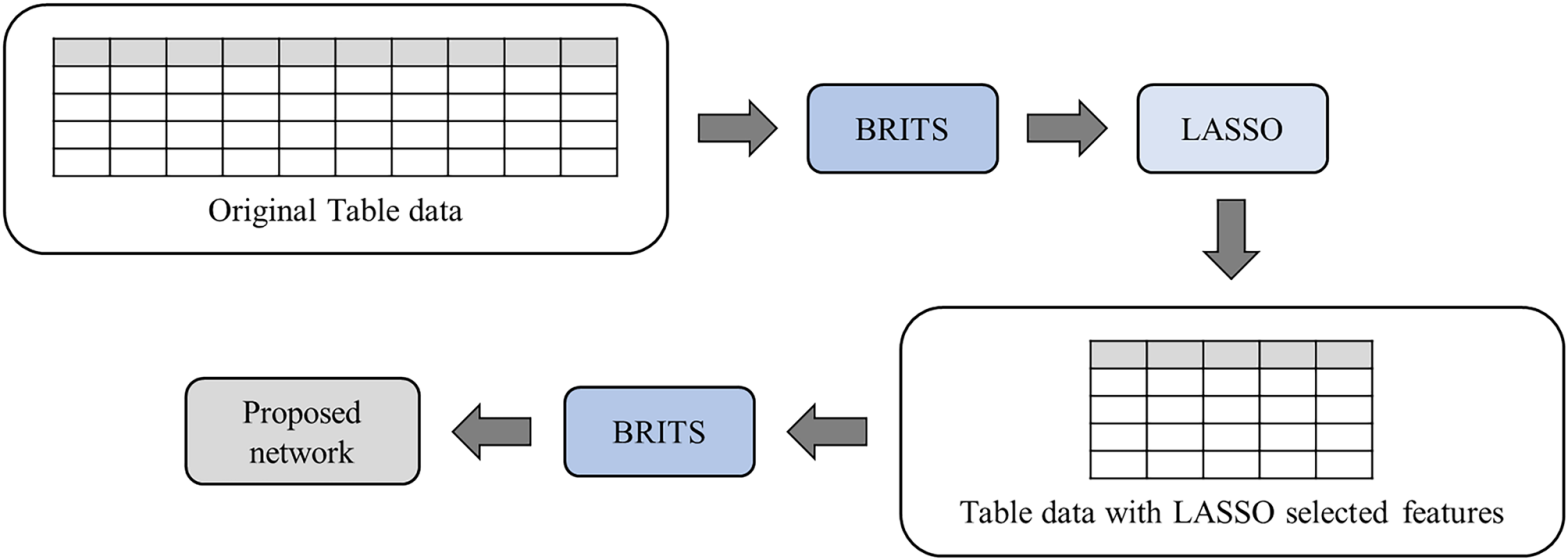
Schematic diagram of the BRITS applications.

BRITS is a powerful algorithm that leverages a bidirectional recurrent neural network (BRNN) to impute missing values in multivariate time series data, taking advantage of feature means, standard deviations, and trends^34^. Notably, BRITS can perform imputation and classification or regression tasks concurrently, acting as a versatile multi-task learning algorithm. In our study, we utilized BRITS imputation twice. First, we applied BRITS imputation before the LASSO feature selection step to mitigate any potential biases and to ensure proper selection of appropriate features for the model. Second, we applied BRITS imputation before training the proposed network, but only for the selected 48 features instead of the entire feature set. The BRITS algorithm treats missing values as variables within the bidirectional RNN graph, performing missing value imputation and classification/regression applications simultaneously. Consequently, the combination of variables can impact the accuracy of imputation. Thus, the second application of the BRITS imputation helps to exclude less important features from the process, allowing only relevant features to be incorporated into the progressive self-transfer architecture. Figure 4 presents the schematic diagram of the BRITS applications as described above.

### Progressive self-transfer framework

The proposed progressive self-transfer framework was implemented using PyTorch libraries and trained on a Linux Intel(R) Core (TM) i7-9700 CPU with a Nvidia GeForce RTX 2070 SUPER GPU environment. The network was trained and tested with a batch size of 32, a dropout rate of 0.2, and binary cross entropy loss with sigmoid function as the loss function. The experiments were repeated 10 times to obtain the means and standard deviations of the results.

**Figure 5:**
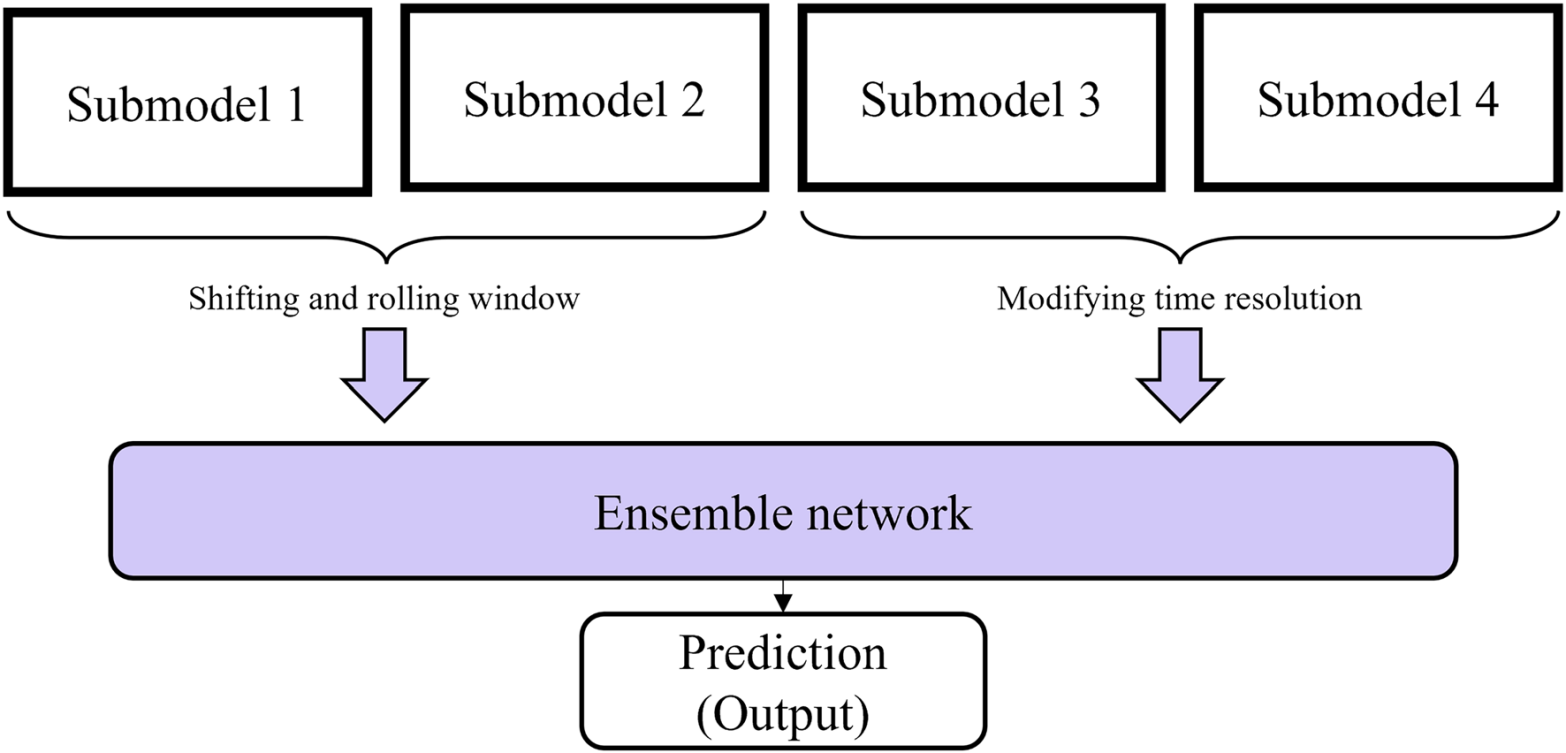
The overall architecture of the progressive self-transfer network. Submodels 1–4 are the examples of the downstream classifiers.

Figure 5 illustrates the proposed framework, which includes multiple submodels and utilizes an ensemble method to soft vote the classification scores of the sub-classifiers for final prediction. The submodels are multilayered LSTM-based self-transfer networks that train and test in a sequential manner. The introduction of the multilayered LSTM aims to fully capture and address the dynamic temporal patterns in the data. The forget, input, and output gates of LSTM control the unit cell, effectively resolving the vanishing gradient and short-term memory issues of traditional RNNs. The forget gate decides which information from the previous cell state can be discarded. The input gate determines which new information should be added to the cell state. Finally, the output gate identifies the most significant information from the current cell state to determine the value of the next hidden state. The purpose of the submodels is to capture the dynamic characteristics of the time series data, such as the varying label imbalance among the time steps. The framework is designed to process training and testing data in a sequential manner, as humans perceive time series data not only in sequence but also as a whole simultaneously. The progressive self-transfer learning scheme allows for the former learning step to provide information to the latter learning process in a sequential manner. Meanwhile, baseline models were introduced to verify and explain the performance of the proposed model. To fully consider the uniqueness of each submodel and track the improvement in evaluation metrics, non-progressive self-transfer networks were used as a baseline model for the four progressive self-transfer networks. Additionally, we compared our experimental results against three well-known recurrent neural networks: LSTM, GRU, and RNN. These models can capture temporal patterns in sequential data. RNN is a traditional recurrent model in which the output of the former step becomes the input of the current stage, especially suited for data with relatively short time-steps. However, as the sequence length of the data increases, issues like gradient vanishing or exploding may occur at times. GRU is similar to the LSTM described above; however, it consists of two gates: an update gate and a reset gate. The update gate determines how much information from the previous time step is updated to the current state, while the reset gate decides which information to disregard.

**Figure 6:**
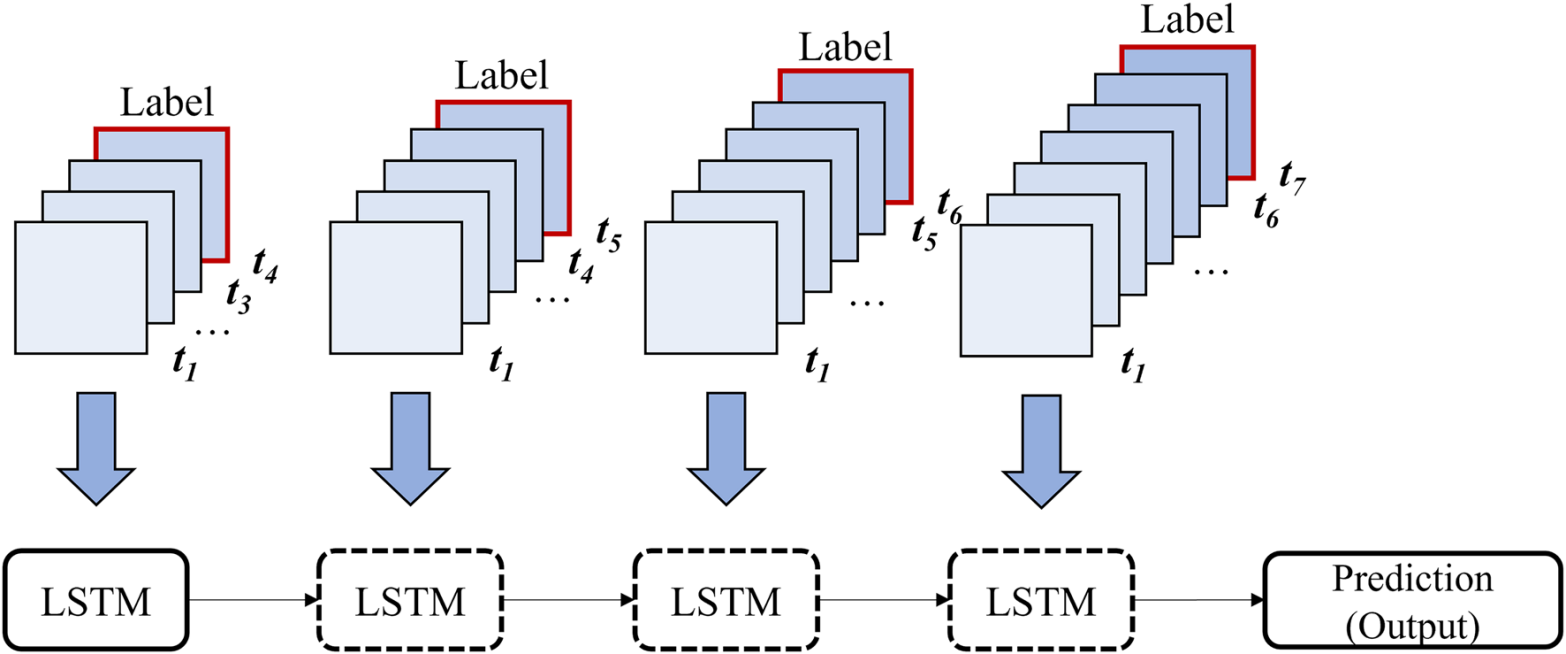
An example of the progressive self-transfer submodel. The model is trained and tested in a sequential flow. The LSTM boxes with dotted lines represent the learning steps the weights of which are transferred from the former learning step.

Figure 6 illustrates an example of the progressive self-transfer submodel used in the study. The network has 48 input dimensions, which were selected in the LASSO feature selection step. The model consists of sequential tasks, where the first task is to predict whether the participants will develop diabetes at the time step *t*_*4*_ using the data from the time steps *t*_*1*_ to *t*_*3*_. The second task is to predict the same at the time step *t*_*5*_ using the data from the time steps *t*_*1*_ to *t*_*4*_. The sequential progress is achieved by gradually adding time steps, and the best epoch's weight is transferred to the consequent tasks via weight initialization. The preprocessing methods shifting and rolling window and modifying time resolution will be discussed in the next section.

Self-supervised learning and transfer learning are the key concepts behind the proposed network. Self-supervised learning is a technique where the labels are acquired from the data itself, and the data is partially used to predict the other parts of the data^35^. A pretext task is a task designed to improve the performance of the target prediction. In this study, the pretext task was the binary prediction of diabetes, formulated according to two time series data processing methods. Self-supervised sequences were carried out using transfer learning, which efficiently improved the performance of the pretext tasks over time. Only some finetuning was necessary without changes to the model architecture. Furthermore, the proposed framework increased the total number of model training iterations compared to single whole-data training, enabling the model to better perceive the self-data. In other words, the progressive self-transfer network transferred knowledge in sequence to capture high-level information of the self-data.

### Time series data preprocessing: shifting and rolling window

**Figure 7:**
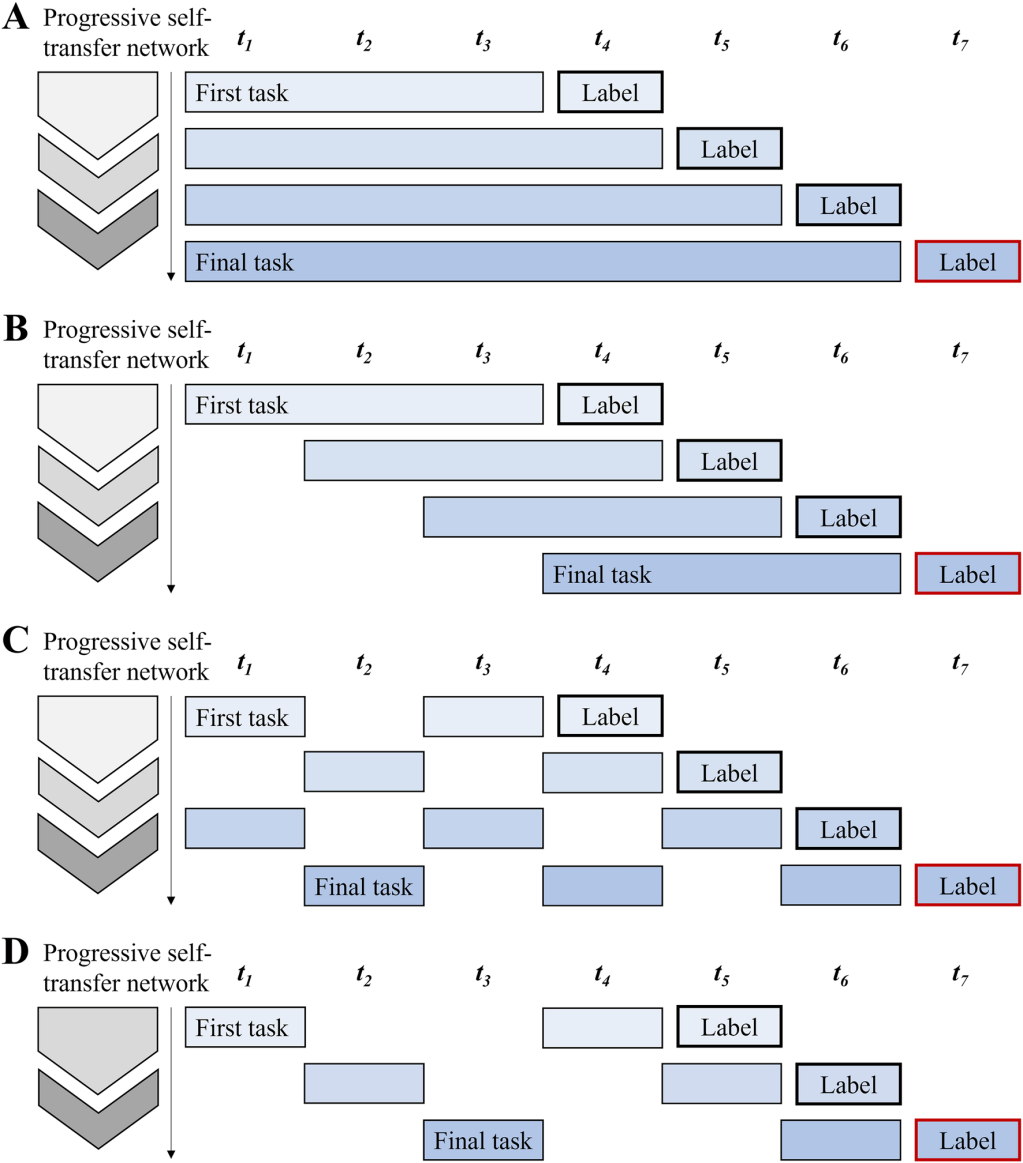
A schematic illustration of the submodels employed in the proposed progressive self-transfer network. Each submodel consists of sequential tasks, and modified time series data is used as input of the network in a specific order. (**A**) Submodel 1. (**B**) Submodel 2. (**C**) Submodel 3. (**D**) Submodel 4.

Following the feature-level data preprocessing, we performed data preprocessing at the time-step level to enable sequential inputs for the proposed model framework. As the KoGES Ansan and Ansung study provides discrete time series data, we had explicit and repeated time intervals by the inspections. Based on a time window interval of two years, we adopted a modification technique that involves changing the window size or rolling the time window gradually. The first method we used is the shifting and rolling window approach. We applied this method in submodels 1 and 2, as shown in schematic diagrams A and B in Fig. 7. In submodel 1, the length of the time window gradually increased as the tasks proceeded. Training began with a window size of six years, which refers to the first three time steps, since the length of a single window is two years. For submodel 2, the length of the time window was fixed, and it was rolled as the tasks proceeded. Consequently, sequential inputs were progressively provided by the time series data. The following formula defines the model input for each sequence, which is formed by shifting and rolling the time window of the original data:

1 $$I = \left\{ {\left( {d_{x} , d_{y} } \right) {|} d_{{x_{i} }} \in X; d_{{y_{i} }} \in Y; i = 1, 2, \ldots , p} \right\}$$

2 $$X_{submodel 1} = \left\{ {d_{x} {|} d_{{x_{i} }} = \mathop \sum \limits_{t = 1}^{i - n + 1} x_{t} , 1 \le i \le N - n} \right\}$$

3 $$X_{submodel 2} = \left\{ {d_{x} | d_{{x_{i} }} = \mathop \sum \limits_{t = 1}^{i - n + 1} x_{t} - \mathop \sum \limits_{t = 1}^{i - 2n + 1} x_{t} , 1 \le i \le N - n} \right\}$$

4 $$Y = \left\{ {d_{y} {|} d_{{y_{i} }} = y_{i + n} , 1 \le i \le N - n} \right\}$$

The input data of the model, represented by *I*, is defined in Eq. (1), which consists of the pairs of $d_{x}$ and $d_{y}$. Each of the $d_{{x_{i} }}$ and $d_{{y_{i} }}$ are feature and label data of participants in an appropriate format for training sequence *i*. These pairs of data are composed of $x_{t}$ and $y_{t}$, which represent data in the time step *t*. The sequential training is performed *p* times, and *X* and *Y* are the generalized expressions of $d_{{x_{i} }}$ and $d_{{y_{i} }}$. However, since the time series data preprocessing methods of submodel 1 and submodel 2 are different, the expressions of *X* are diversified as shown in Eqs. (2) and Eq. (3). Here, *N* represents the number of total time steps in the data, and *n* represents the width of the initial time window, which is increased or rolled over the training sequence.

### Time series data preprocessing: modifying time resolution

Modifying time resolution is another method for creating sequential inputs in time series data preprocessing. This approach involves zooming in and out of the data to inspect information from different perspectives. In this study, as the data was collected every two years, the minimum time resolution is two years. To reflect both micro and macro aspects of the data, we doubled or tripled the time resolution of the dataset. Submodel 3 (Fig. 7c) doubles the time resolution, simulating window intervals of four years and creating four sequential inputs. Submodel 4 (Fig. 7d) triples the time resolution, simulating window intervals of six years and creating three task inputs. The following formula is used to define the sequential input, which is developed by varying the time resolution of the original data:

5 $$X = \left\{ {d_{x} {|} d_{{x_{i} }} = \mathop \sum \limits_{s = 0} x_{{i + \left( {1 - s} \right)\left( {w + 1} \right)}} , 1 \le i \le N - w - 2} \right\}$$

6 $$Y = \left\{ {d_{y} {|} d_{{y_{i} }} = y_{i + w + 2} , 1 \le i \le N - w - 2} \right\}$$

The structure of the input *I* follows the same formula as mentioned earlier. However, the variables *X* and *Y* are dependent on the skipping series of the time window intervals *w*, as shown in Eqs. (5) and Eq. (6). ‘Skipping series’ refers to an input dataset configured to exclude data from specific time windows, in accordance with changes in time resolution. For instance, if *w* = 1, this sets the skipping interval at 1. Consequently, the skipping series is created by omitting every other data point, starting from the end and moving backwards until the first time-step is reached. The value of *w* is specific to each submodel, with submodel 3 using a skipping interval of 1 and submodel 4 using a skipping interval of 2. The value of *w* is determined based on the total number of time steps in the data.

### Ensemble

The final step of the proposed framework involves ensembling the classification scores of the submodels. This step aims to compensate for the imbalanced labels and variable feature properties and to prevent overfitting of the prediction results. Since each submodel has its own unique learning strategy and perspective on the data, we concatenate these perspectives via soft voting to calculate the mean probability. The weights of all perspectives are considered equal. To find the best combination of multiple learners, we ensemble diverse combinations of the submodels (submodel 1–4) to find the best evaluation performances.

### Evaluation

In this study, a five-fold cross-validation was used to validate the model and to prevent overfitting. The dataset of 3379 participants was divided into five folds based on the binary labels of the last time step. Each fold was used as a validation set in consecutive order, and the mean metrics of the validation sets were used to evaluate the model performance. Accuracy, AUC, recall, precision, and F1 score were considered for the overall binary classification evaluation. All models, including the non-progressive single self-transfer network and ensemble results, were compared based on these five metrics. In the submodel performance evaluation, AUC was considered the most important metric to determine the best epoch and continue training in sequence, as it reflects the classification performance for all classes. The weight of the best epoch was then transferred to the following similar tasks based on AUC.

### Supplementary Information

Supplementary Tables.

## Data Availability

The KoGES dataset utilized in this research is open to the public and available via registration to the Korean National Institute of Health (https://nih.go.kr/ko/main/contents.do?menuNo=300578).
