# Supplementary material for: Advancing diabetes prediction with a progressive self-transfer learning framework for discrete time series data
Source: Sci Rep. 2023 Nov 29;13:21044. doi: 10.1038/s41598-023-48463-0 (PMC10687240; doi:10.1038/s41598-023-48463-0)
Supplement: Supplementary file 1 — Supplementary Tables. [file 41598_2023_48463_MOESM1_ESM.pdf]

Supplementary information for:

Advancing Diabetes Prediction with a Progressive Self-Transfer Learning Framework for Discrete Time Series Data

Heeryung Lim[1], Gihyeon Kim[2], Jang-Hwan Choi[1*]

[1]Division of Mechanical and Biomedical Engineering, Graduate Program in System Health Science and Engineering, Ewha Womans University, Seoul 03760, Korea
[2]Department of Computational Medicine, Graduate Program in System Health Science and Engineering, Ewha Womans University, Seoul 03760, Korea.

[*]Correspondence: choij@ewha.ac.kr

This file includes:

Table S1
Table S2

Table S1. Descriptive statistics of the LASSO selected 48 features.

Attribute code	Attribute code	t_7 label 0 (n = 2824)		t_7 label 1 (n = 555)		All (n = 3379)	
		t_1	t_6	t_1	t_6	t_1	t_6
AGE	Age, years	54.5 ± 8.01	64.46 ± 8.04	54.47 ± 7.83	64.46 ± 7.84	54.49 ± 7.98	64.46 ± 8.01
ALT_ORI	ALT (SGPT), IU/L	22.35 ± 26.04	22.15 ± 14.13	26.48 ± 15.27	27.76 ± 14.26	23.03 ± 24.64	23.07 ± 14.3
AST_ORI	AST (SGOT), IU/L	24.89 ± 16.92	25.25 ± 8.92	25.78 ± 9.88	27.87 ± 13.18	25.04 ± 15.98	25.68 ± 9.79
BDCEXFT	Fat-free mass, kg	46.15 ± 8.25	43.43 ± 8.29	47.71 ± 8.6	44.63 ± 8.49	46.4 ± 8.33	43.62 ± 8.33
BDCFT	Body fat, kg	15.96 ± 5.06	18.55 ± 5.84	18.97 ± 5.51	21.64 ± 6.53	16.46 ± 5.25	19.06 ± 6.07
BDCINWT	Intracellular fluid, L	21.44 ± 3.93	19.7 ± 3.87	22.24 ± 4.09	20.27 ± 3.97	21.57 ± 3.97	19.79 ± 3.9
BDCMSC	Body muscle mass, kg	43.56 ± 7.89	41.02 ± 7.89	45.05 ± 8.22	42.16 ± 8.07	43.81 ± 7.96	41.21 ± 7.93
BDCPRT	Body protein, kg	11.63 ± 2.11	8.51 ± 1.67	12.03 ± 2.2	8.75 ± 1.72	11.7 ± 2.13	8.55 ± 1.68
BDCWT	Body water, L	31.93 ± 5.78	31.99 ± 6.12	33.02 ± 6.03	32.88 ± 6.26	32.11 ± 5.84	32.13 ± 6.15
BDFTR	Body fat rate, %	25.67 ± 6.98	29.88 ± 7.61	28.43 ± 6.81	32.47 ± 7.53	26.12 ± 7.03	30.3 ± 7.65
BMI	BMI, kg/m^2	24.17 ± 2.86	24.25 ± 3.01	26.02 ± 3.02	25.96 ± 3.37	24.47 ± 2.96	24.53 ± 3.14
BPLIE1D	Lie - DBP (1st), mmHg	74.97 ± 9.74	72.47 ± 9.39	77.83 ± 9.82	74.2 ± 9.47	75.44 ± 9.81	72.76 ± 9.43
BPLIE2S	Lie - SBP (2nd), mmHg	113.49 ± 15.26	117.36 ± 15.02	117.9 ± 15.33	120.98 ± 14.04	114.21 ± 15.36	117.96 ± 14.92
BPSIT1LD	Sit - Left arm DBP, mmHg	76.96 ± 10.45	75.94 ± 9.95	79.82 ± 10.05	77.53 ± 9.72	77.43 ± 10.44	76.2 ± 9.93
BPSIT1LS	Sit - Left arm SBP, mmHg	113.86 ± 16.08	119.9 ± 16.64	118.24 ± 15.68	123.66 ± 16.28	114.58 ± 16.09	120.52 ± 16.63
BPSIT1RD	Sit - Right arm DBP, mmHg	76.84 ± 10.31	75.27 ± 9.6	79.55 ± 9.77	76.5 ± 9.25	77.28 ± 10.27	75.47 ± 9.55
BPSIT1RS	Sit - Right arm SBP, mmHg	114.2 ± 15.88	119.27 ± 16.31	118.59 ± 15.5	122.74 ± 15.29	114.92 ± 15.9	119.84 ± 16.19
BPSITL	Sit - Left tactile SBP, mmHg	116.07 ± 16.03	118.97 ± 15.71	120.03 ± 15.27	122.55 ± 15.65	116.72 ± 15.97	119.56 ± 15.75
BPSITR	Sit - Right tactile SBP, mmHg	115.95 ± 15.96	118.57 ± 15.54	120.03 ± 15.43	122.23 ± 14.61	116.62 ± 15.95	119.17 ± 15.45
BUN_ORI	BUN, mg/dL	15.57 ± 4.17	16.53 ± 4.57	15.61 ± 3.95	16.4 ± 5.09	15.57 ± 4.13	16.51 ± 4.66
CREATININE_ORI	Creatinine, mg/dL	0.96 ± 0.15	0.96 ± 0.19	0.97 ± 0.16	1 ± 0.36	0.96 ± 0.15	0.96 ± 0.23
CRP	C-Reactive protein, mg/dL	1.32 ± 3.4	1.41 ± 4	1.71 ± 2.21	1.6 ± 2.74	1.39 ± 3.24	1.44 ± 3.82
GLU60_ORI	Glucose (1-h OGTT), mg/dL	140.93 ± 38.96	154.42 ± 41.4	191.35 ± 47.01	217.93 ± 42.72	149.21 ± 44.5	164.85 ± 47.81
HB	Hemoglobin, g/dL	13.69 ± 1.54	13.31 ± 1.33	13.94 ± 1.54	13.46 ± 1.36	13.73 ± 1.54	13.33 ± 1.34
HCT	Hematocrit, %	41.06 ± 4.28	41.26 ± 4.05	41.79 ± 4.28	41.56 ± 4.17	41.18 ± 4.29	41.31 ± 4.07
HDL_ORI	HDL-Cholesterol, mg/dL	45.01 ± 10.36	47.07 ± 11.99	41.96 ± 9.73	42.72 ± 10.85	44.51 ± 10.32	46.35 ± 11.92
HEIGHT	Height, cm	160.07 ± 8.5	159.46 ± 8.82	159.83 ± 8.73	159.29 ± 9.03	160.03 ± 8.54	159.43 ± 8.85
HIP1	Hip circumference (1st), cm	91.54 ± 5.08	93.43 ± 5.79	93.42 ± 5.43	95.49 ± 6.63	91.85 ± 5.18	93.77 ± 5.99
HIP2	Hip circumference (2nd), cm	91.55 ± 5.07	93.42 ± 5.79	93.42 ± 5.42	95.48 ± 6.63	91.86 ± 5.18	93.76 ± 5.99
HIP3	Hip circumference (3rd), cm	91.55 ± 5.07	93.43 ± 5.79	93.43 ± 5.43	95.49 ± 6.63	91.86 ± 5.18	93.76 ± 5.99
INS0	Insulin (fasting), μIU/㎖	7.24 ± 3.45	8.6 ± 3.67	8.94 ± 4.15	10.99 ± 5.3	7.52 ± 3.63	8.99 ± 4.08
INS120	Insulin (2-h OGTT), μIU/㎖	23.99 ± 24.63	32.06 ± 30.52	34.43 ± 35.46	41.81 ± 34.96	25.7 ± 26.98	33.66 ± 31.49
INS60	Insulin (1-h OGTT), μIU/㎖	31.05 ± 28.17	35.31 ± 27.03	40.03 ± 37.16	34.87 ± 26.85	32.52 ± 30.01	35.24 ± 27
OBDG	Obesity degree, %	116.76 ± 14.88	113.14 ± 14.44	125.88 ± 15.83	121.06 ± 16.16	118.26 ± 15.41	114.44 ± 15.03
PH_U	Urine (16) - pH	5.93 ± 0.86	5.8 ± 0.8	5.81 ± 0.82	5.73 ± 0.83	5.91 ± 0.85	5.79 ± 0.81
PLAT	Plaltlet, 10^3/μL	241.95 ± 55.37	241.16 ± 56.4	252.55 ± 62.11	249.17 ± 60.98	243.69 ± 56.66	242.48 ± 57.25
PLLIE1	Lie - pulse rate (1st), bpm	63.66 ± 6.61	62.42 ± 7.27	64.37 ± 6.55	63.72 ± 8.25	63.78 ± 6.6	62.63 ± 7.45
RBC	Blood - R.B.C 10^6/μL	4.4 ± 0.43	4.33 ± 0.44	4.5 ± 0.44	4.41 ± 0.47	4.41 ± 0.43	4.35 ± 0.44
SUB2	Subscapular (2nd), mm	22.13 ± 8.15	28.06 ± 8.47	25.41 ± 8.58	30.74 ± 8.01	22.67 ± 8.31	28.5 ± 8.45
SUB3	Subscapular (3rd), mm	22.13 ± 8.14	23.43 ± 7.83	25.42 ± 8.58	26.25 ± 7.92	22.67 ± 8.3	23.89 ± 7.91
SUP1	Suprailiac (1st), mm	31.83 ± 10.74	28.85 ± 7.31	36.67 ± 11.11	31.48 ± 7.31	32.63 ± 10.95	29.28 ± 7.37
SUP2	Suprailiac (2nd), mm	31.85 ± 10.75	27.18 ± 7.06	36.69 ± 11.11	29.83 ± 7.32	32.65 ± 10.95	27.61 ± 7.17
SUP3	Suprailiac (3rd), mm	31.85 ± 10.74	28.76 ± 7.27	36.7 ± 11.1	31.43 ± 7.3	32.65 ± 10.95	29.2 ± 7.34
TCHL_ORI	Total cholesterol, mg/dL	191.12 ± 33.58	189.05 ± 34.05	195.12 ± 33.51	180.38 ± 35.56	191.78 ± 33.6	187.63 ± 34.45
TG_ORI	Triglyceride, mg/dL	125.52 ± 82.22	126.1 ± 78.3	169.21 ± 125.61	163.37 ± 108.03	132.7 ± 92.19	132.22 ± 85.02
WAIST1	Waist circumference (1st), cm	82.95 ± 8.58	86.02 ± 9.03	87.55 ± 8.72	90.59 ± 9.52	83.71 ± 8.77	86.77 ± 9.27
WBC	Blood - W.B.C, 10^3/μL	6.06 ± 1.74	5.22 ± 1.41	6.65 ± 1.82	5.91 ± 1.63	6.15 ± 1.77	5.34 ± 1.47
WEIGHT	Weight, kg	61.97 ± 9.45	61.8 ± 10.04	66.5 ± 10.16	66.1 ± 10.99	62.71 ± 9.71	62.51 ± 10.33

Table S2. LASSO coefficients of the selected 48 features.

Attribute code	Description	LASSO coefficient
AGE	Age, years	0.005084
ALT_ORI	ALT (SGPT), IU/L	0.007943
AST_ORI	AST (SGOT), IU/L	-0.007553
BDCEXFT	Fat-free mass, kg	0.005135
BDCFT	Body fat, kg	0.102842
BDCINWT	Intracellular fluid, L	0.042784
BDCMSC	Body muscle mass, kg	0.000895
BDCPRT	Body protein, kg	-0.046126
BDCWT	Body water, L	0.013967
BDFTR	Body fat rate, %	-0.016367
BMI	BMI, kg/m^2	-0.056062
BPLIE1D	Lie - DBP (1st), mmHg	-0.001038
BPLIE2S	Lie - SBP (2nd), mmHg	0.006522
BPSIT1LD	Sit - Left arm DBP, mmHg	0.001151
BPSIT1LS	Sit - Left arm SBP, mmHg	0.012864
BPSIT1RD	Sit - Right arm DBP, mmHg	-0.014911
BPSIT1RS	Sit - Right arm SBP, mmHg	-0.002204
BPSITL	Sit - Left tactile SBP, mmHg	-0.008607
BPSITR	Sit - Right tactile SBP, mmHg	0.000581
BUN_ORI	BUN, mg/dL	-0.018122
CREATININE_ORI	Creatinine, mg/dL	0.599667
CRP	C-Reactive protein, mg/dL	0.002122
GLU60_ORI	Glucose (1-h OGTT), mg/dL	0.032466
HB	Hemoglobin, g/dL	-0.110856
HCT	Hematocrit, %	-0.006471
HDL_ORI	HDL-Cholesterol, mg/dL	-0.011587
HEIGHT	Height, cm	-0.045274
HIP1	Hip circumference (1st), cm	-0.001803
HIP2	Hip circumference (2nd), cm	-0.00047
HIP3	Hip circumference (3rd), cm	-0.000205
INS0	Insulin (fasting), μIU/㎖	0.048585
INS120	Insulin (2-h OGTT), μIU/㎖	0.003073
INS60	Insulin (1-h OGTT), μIU/㎖	-0.014758
OBDG	Obesity degree, %	-0.010646
PH_U	Urine (16) - pH	-0.019102
PLAT	Plaltlet, 10^3/μL	0.000999
PLLIE1	Lie - pulse rate (1st), bpm	-0.001344
RBC	Blood - R.B.C 10^6/μL	0.05389
SUB2	Subscapular (2nd), mm	-0.031222
SUB3	Subscapular (3rd), mm	0.053911
SUP1	Suprailiac (1st), mm	-0.023569
SUP2	Suprailiac (2nd), mm	-0.011968
SUP3	Suprailiac (3rd), mm	0.014935
TCHL_ORI	Total cholesterol, mg/dL	-0.003325
TG_ORI	Triglyceride, mg/dL	0.002108
WAIST1	Waist circumference (1st), cm	0.002054
WBC	Blood - W.B.C, 10^3/μL	0.041984
WEIGHT	Weight, kg	0.013548
